# Changes in radiomic and radiologic features in meningiomas after radiation therapy

**DOI:** 10.1186/s12880-023-01116-0

**Published:** 2023-10-19

**Authors:** Sang Won Jo, Eun Soo Kim, Dae Young Yoon, Mi Jung Kwon

**Affiliations:** 1 Department of Radiology, Dongtan Sacred Heart Hospital, Hallym University College of Medicine, Hwaseong-si, Gyeonggi-do South Korea; 2 Department of Radiology, Hallym University Sacred Heart Hospital, Hallym University College of Medicine, 22, Gwanpyeong-ro 170beon-gil, Dongan-gu, Anyang-si, 14068 Gyeonggi-do Republic of Korea; 3 Department of Radiology, Kangdong Sacred Heart Hospital, Hallym University College of Medicine, Seoul, South Korea; 4 Department of Pathology, Hallym University Sacred Heart Hospital, Hallym University College of Medicine, Anyang-si, Gyeonggi-do South Korea

**Keywords:** Radiomics, Meningioma, Radiation therapy, Radiology, MRI

## Abstract

**Objectives:**

This study evaluated the radiologic and radiomic features extracted from magnetic resonance imaging (MRI) in meningioma after radiation therapy and investigated the impact of radiation therapy in treating meningioma based on routine brain MRI.

**Methods:**

Observation (n = 100) and radiation therapy (n = 62) patients with meningioma who underwent MRI were randomly divided (7:3 ratio) into training (n = 118) and validation (n = 44) groups. Radiologic findings were analyzed. Radiomic features (filter types: original, square, logarithm, exponential, wavelet; feature types: first order, texture, shape) were extracted from the MRI. The most significant radiomic features were selected and applied to quantify the imaging phenotype using random forest machine learning algorithms. Area under the curve (AUC), sensitivity, and specificity for predicting both the training and validation sets were computed with multiple-hypothesis correction.

**Results:**

The radiologic difference in the maximum area and diameter of meningiomas between two groups was statistically significant. The tumor decreased in the treatment group. A total of 241 series and 1691 radiomic features were extracted from the training set. In univariate analysis, 24 radiomic features were significantly different (P < 0.05) between both groups. Best subsets were one original, three first-order, and six wavelet-based features, with an AUC of 0.87, showing significant differences (P < 0.05) in multivariate analysis. When applying the model, AUC was 0.76 and 0.79 for the training and validation set, respectively.

**Conclusion:**

In meningioma cases, better size reduction can be expected after radiation treatment. The radiomic model using MRI showed significant changes in radiomic features after radiation treatment.

**Supplementary Information:**

The online version contains supplementary material available at 10.1186/s12880-023-01116-0.

## Introduction

Meningioma is the most common primary brain tumor in adults and is mostly considered benign by the World Health Organization (WHO) histopathological criteria (WHO Grade 1) [1, 2]. Medical imaging plays a fundamental part in the differential diagnosis and treatment plan of central nervous system tumors, including meningiomas. If a meningioma is judged as small, stable, and benign, the wait-and-see method, i.e., observing with a long-term follow-up, may be an ideal and cost-effective option. Conversely, if the growth rate is high or malignancy is suspected on genotyping, early surgical resection is strongly recommended, even if the size is small.

Radiotherapy is sometimes the primary treatment for meningioma. It is often performed if surgery is contraindicated because of tumor proximity to critical nerves or vessels or due to the patient’s concerns about the surgery or poor health. Additionally, radiotherapy is performed as adjuvant therapy to destroy any remaining tumor cells and prevent recurrence if the meningioma has been incompletely removed or in high-grade and/or recurrent tumors.

The radiographic appearance of a tumor can be described using quantitative and qualitative measures. Radiomics is an emerging field of quantitative imaging focused on leveraging large sets of imaging features to create an atlas [3, 4] that fosters the automatic, reproducible, and unbiased assessment of active clinical cases [1, 5]. It provides an objective, quantitative approach to interpreting imaging data rather than subjective, qualitative interpretations that rely on relatively limited human visual observations [6, 7]. In contrast, radiographic features are tumor traits (e.g., signal intensity [SI], bony invasion, necrosis) assessed visually by radiologists.

Information generated via radiomics analyses provides radiological to histopathological tumor information that cannot be perceived visually [6]. This information also offers a technological basis for its applications in diagnosis, treatment, and prognosis [6]. To our knowledge, no study has evaluated the changes in meningiomas after radiation therapy based on radiomic or texture features analysis to date. This study investigated the changes in radiomic and radiologic features of meningiomas following radiation therapy using routine magnetic resonance imaging (MRI).

## Materials and methods

The CLEAR checklist was used for guiding the reporting of current study and is presented in a supplementary Table 1 [8].

### Patient selection

**Fig. 1:**
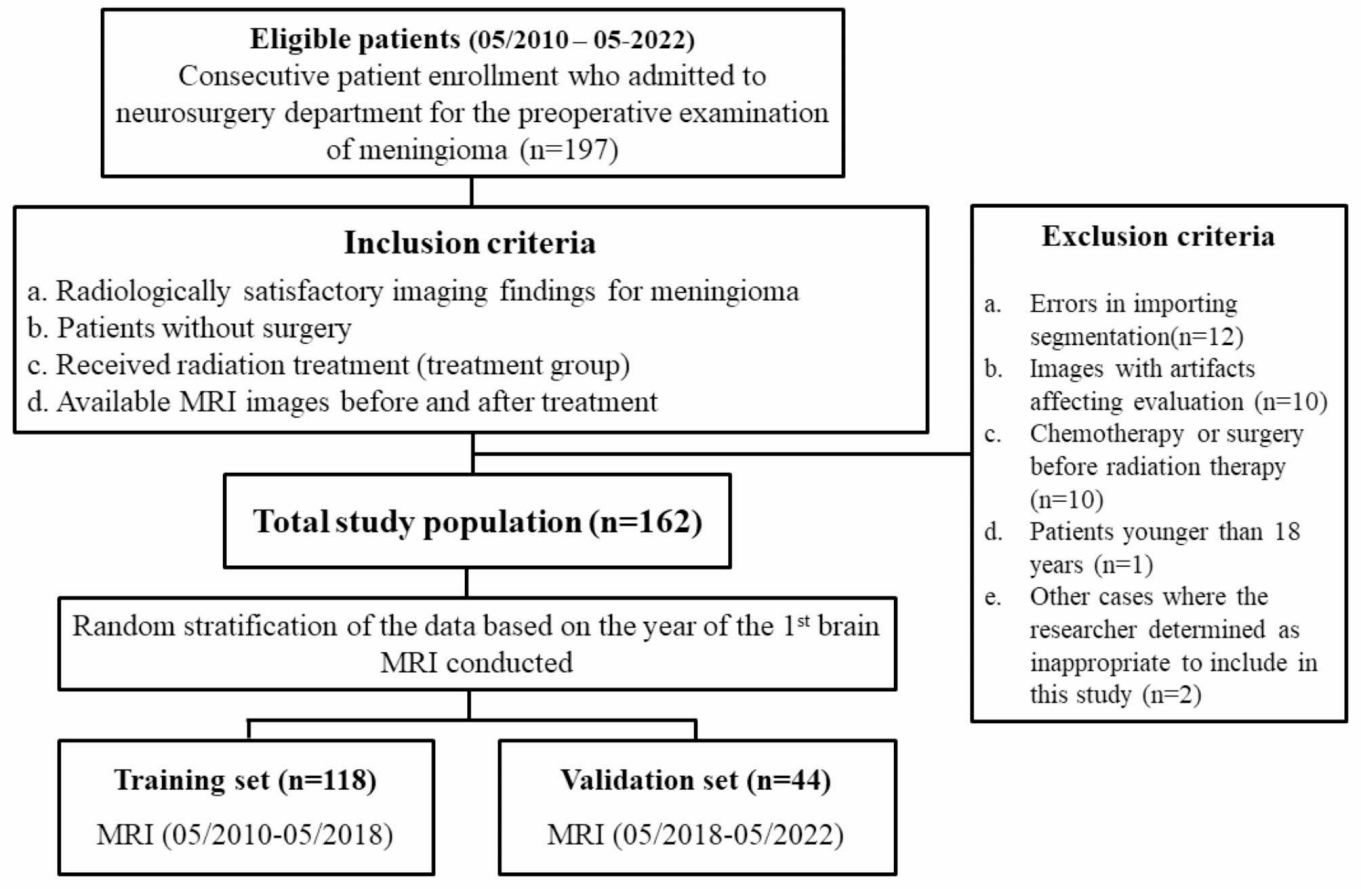
Flowchart shows selection of the study population

This retrospective study was approved by the institutional review board of Hallym University Sacred Heart hospital (Approval No. 2021-10-016), and the need to obtain informed consent was waived. We included 197 patients admitted to our neurosurgery department for the preoperative examination of meningiomas between May 2010 and May 2022. The patient flowchart in Fig. 1 shows selection of the study population. The inclusion criteria were: (1) radiologically satisfactory imaging findings for meningiomas, (2) patients without surgery, (3) patients who received radiation therapy (treatment group), and (4) available MRI images before and after treatment. We excluded 35 patients for the following reasons: (1) errors in importing segmentation (n = 12), (2) patient images with artifacts affecting evaluation (n = 10), (3) patients who had received chemotherapy or surgery before radiation therapy (n = 10), (4) patients younger than 18 years (n = 1), and (5) other cases where the researcher determined that participation in this study was not appropriate (n = 2). Eventually, 162 eligible patients were selected for analysis and divided into two cohorts: a training set (n = 118) and a validation set (n = 44) through random stratified sampling to ensure an even distribution. To perform this random stratified sampling for a uniform distribution, we segregated the data based on the year of the first brain MRI conducted at the hospital. Patients who underwent their MRI between May 2010 and May 2018 were included in the training set, while those who had their MRI between June 2018 and May 2022 were assigned to the validation set. Clinical information, including age at diagnosis, gender, follow-up period, post-treatment period, total radiation dose, and tumor location, were retrieved from our institution’s electronic medical records. The patients’ medical record review was completed in May 2022.

### Image acquisition

**Fig. 2:**
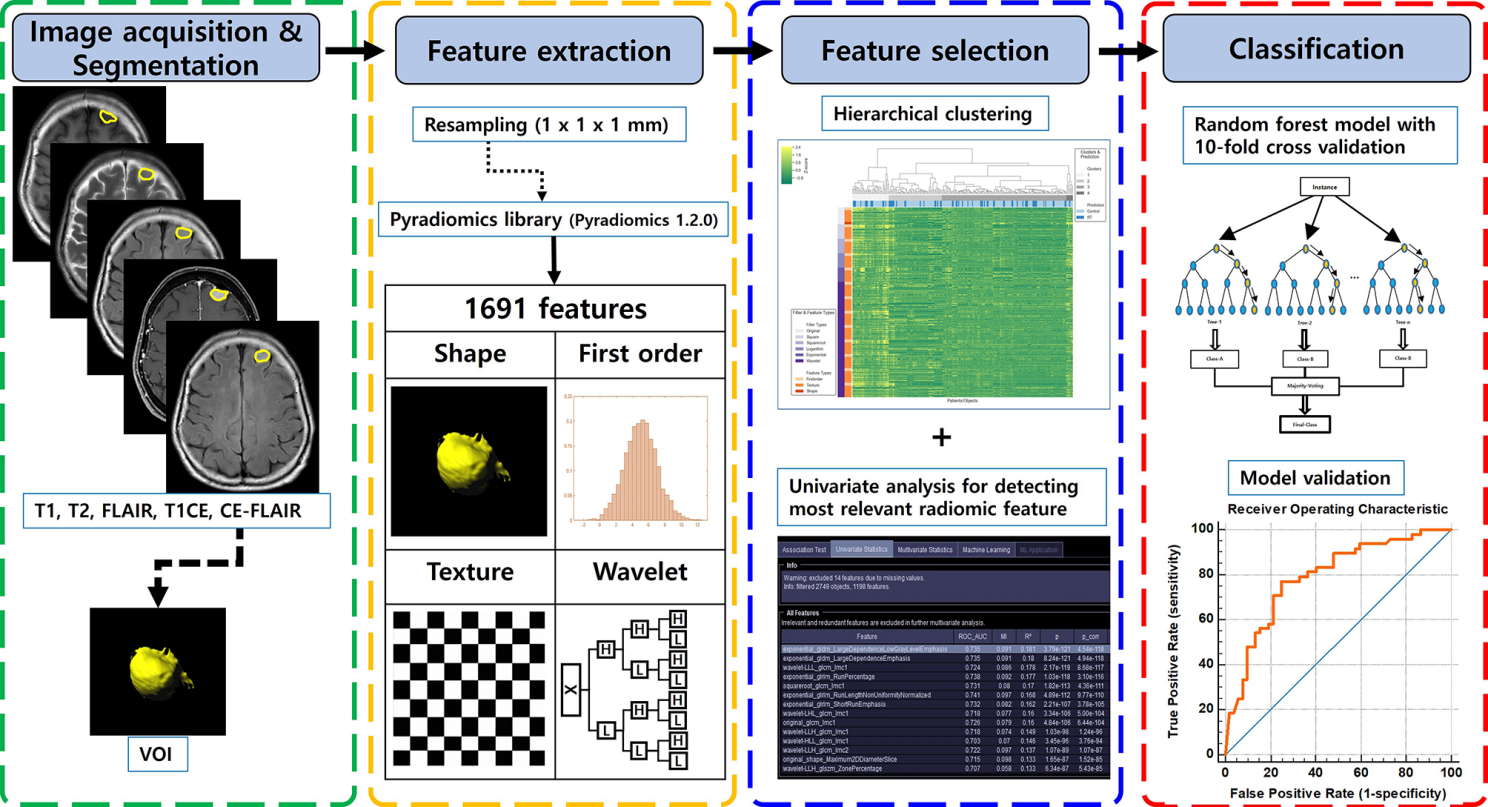
Workflow of the development and testing of a radiomics model. First, lesions were semiautomatically segmented on MRI scans for radiomic analysis. Second, a total of 1691 radiomics features were extracted. Third, in the training phase, the 10 most relevant features were selected with classic minimum redundancy maximum relevance. The random forest (RF) model was built and validated with the 10-fold cross-validation method. Fourth, in the test phase, the RF model was tested with a validation test set. AUC = area under the receiver operating characteristic curve

Images were acquired using 3.0-T MRI units (Ingenia Elition X or Achieva dStream, Philips Medical Systems; Skyra, Siemens Healthcare). A retrospective study was performed on the observation (n = 100) and radiation therapy (n = 62) patients with meningioma who underwent baseline and follow-up MRI from T1-weighted imaging (T1WI), T2-weighted imaging (T2WI), contrast-enhanced (CE)-T1WI, fluid-attenuated inversion recovery (FLAIR) imaging, and CE-FLAIR imaging. Imaging protocols included noncontrast axial fluid-attenuated inversion recovery (FLAIR) and nonenhanced axial T1-weighted spin-echo with flow compensation sequences. After weight-adjusted injection of a gadolinium-based contrast agent at a dose of 0.1 mmol per kilogram, contrast material–enhanced axial T1-weighted spin echo sequences with flow compensation or three-dimensional T1-weighted gradient-echo sequences were performed. Sequence parameters varied among the different MRI units and reflect the heterogeneity of image data in clinical practice. The detailed contents are listed in the Supplementary Tables 2, 3, 4. A total of 400 radiomic and radiologic features were utilized to measure the imaging phenotype by employing random forest machine learning algorithms. A flowchart of the study is shown in Fig. 2. Area under the curve (AUC) and odds ratio (OR) were calculated after multiple-hypothesis correction. Gadolinium-enhanced T1-weighted MR images were acquired for all patients with a slice thickness of 1 mm. The average tumor volume was 24.6 cm^3^ (ranging from 1.9 to 374 cm^3^), and mean margin dose at the 50% isodose line was 14 Gy (ranging from 4 to 28 Gy). Four patients exhibited peritumoral edema on MR images during radiation treatments. Neurological examinations and MR were performed every 6 months for 2 years after radiation therapy. If there was no progression, an MR image was obtained yearly. New MR images were obtained to assess disease status whenever symptoms that could be related to the tumor occurred. We measured the tumor size, contrast enhancement, SI, and longest diameter by drawing the region of interest (ROI) in PACS.

### Measurement

When measuring the tumor in CE-T1WI, ROI was drawn manually in a multipoint measurement method along the tumor edge where the tumor is largest in the axial image. The size was measured in two dimensions, and the longest part was the diameter. The tumor SI on T1WI, T2WI, FLAIR, CE-FLAIR, and CE-T1WI was measured by placing an ROI. In MRI, a round ROI of about 40 mm^2^ was placed around the most homogeneous part of the tumor, and, SI is measured. Another 40 mm^2^ round ROI was measured in the pons for the standardization of SI. The final tumor SI was set as the mean value of the measurements by two neuroradiologists blinded to the patients’ clinical information. Moreover, we observed the presence or absence of the following: cerebrospinal fluid (CSF) cleft, mass effect, intratumoral heterogeneity, skull hyperostosis, multifocality, bony invasion, midline shift, necrosis/hemorrhage, spiculation, cystic component, venous sinus invasion, recurrence.

### Tumor segmentation for radiomic feature extraction

Radiomic features were extracted from the ROIs on T1WI, T2WI, FLAIR, CE-T1WI, and CE-FLAIR images using a software package (syngo.via Frontier, Siemens Healthineers) based on the PyRadiomics library (Pyradiomics 1.2.0 *(*http://www.radiomics.io/pyradiomics.html*)* [9]. ROIs of the visible gross tumor volume were semi-automatically delineated using the “lesion segmentation” function within the software [10]. This process resulted in a 3D ROI contour, with minor adjustments made to prevent beam-hardening artifacts and to account for adjacent soft tissues. A consensus ROI was created by combining both readers’ segmentations and disagreements were resolved through consensus. Both radiologists were blinded to the patients’ clinical information during image analysis. Representative images of ROI segmentations are provided in Fig. 2.

Initially, the ROIs were resampled to have a uniform voxel size of 1 mm. Radiomic features were extracted using PyRadiomics a publicly accessible platform for radiomic features [9], embedded in syngo.via Frontier. This process generated six different categories of features, automatically extracted, yielding a total of 872 features per patient. The detailed information about these features is available publicly at (https://pyradiomics.readthedocs.io/en/latest/).

A random forest–based wrapper algorithm was employed to select relevant radiomics features. The Random Forest assigns importance scores for each feature, requiring minimal parameter adjustment. Feature importance was assessed using the Boruta algorithm [11], which repetitively evaluated all possible feature combinations, ultimately identifying a subset or relevant features that contributed significantly.

AUC and OR were calculated while multiple-hypothesis correction applied. The selection of features was carried out using the classic maximum relevance minimum redundancy (MRMR) method with the R2 difference. MRMR makes sure that the features selected are not only the ones that provide minimum correlation between the input features but also have a high correlation with the output variable. Radiomic features were extracted to characterize tumors, encompassing the six filter types (original, square, logarithm, exponential, wavelet) and three feature types (first order, texture, shape) for each region. In order to comprehensively assess the tumor in multiple dimensions, wavelet transformation, similar to Fourier analysis, was performed on the tumor region in eight directions. This decomposition of the tumor region images yielded high-frequency (H) or low-frequency components (L) components along three directions. Consequently, eight categories of wavelet features, namely HHH, HHL, HLH, LHH, LLL, LLH, LHL, and HLL, were obtained based on their specific decomposition orders.

Intensity features measured the distribution of gray level in the tumor region and were quantified as mean, energy, entropy, variance, skewness, kurtosis, and uniformity. Radiomic features were extracted from the tumor regions in the observation group and radiation therapy MRIs [12, 13].The most significant features were selected from all extracted radiomic features, which were highly relevant to actual classes while reducing redundancy among them. After providing all modified parameters of pre-processing and radiomic feature extraction, all other parameters remained as a default configuration.

### Machine learning and statistical analysis

Patient characteristics within the analysis cohort, including a representative sample of matching variables, were summarized using percentages for categorical variables and means (standard deviation, SD) for continuous variables. Categorical variables were compared using appropriate statistical tests such as the chi-square test or Fisher exact test. Continuous variables were compared using the Mann–Whitney U test. The paired t-test and Wilcoxon test were utilized to compare the location of meningiomas, maximal diameter, and SI between the observation and radiation treatment groups. The Mann–Whitney U test or independent t-test was employed to compare differences between imaging characteristics.

Machine learning methods were employed using a random forest algorithm to construct a high-performance model. Univariate and multivariate analyses were conducted using radiomic feature data to assess their correlation with the ROI of meningiomas. The models were trained on the training sets, and hyperparameter tuning was performed using a 10-fold cross-validation within the training set. The performance of each fine-tuned model was evaluated using the validation set, including metrics such as accuracy, precision, recall, F1 score, and area under the receiver operating characteristic curve (AUROC). A cutoff value of 0.5, was chosen based on the median probability value. The AUROC with highest value was compared pairwise with other models using Delong’s method. All statistical analyses were conducted using SPSS version 24.0. (SPSS Inc., IBM Corp., Chicago, IL, USA). A significance level of P < 0.05 was considered statistically significant.

## Results

### Patients

Study was performed on controls (n = 100) and radiation therapy patients (n = 62) with meningiomas. The clinical characteristics of the study population are summarized in Table 1. The mean patient age was 69 years (range 40–98 years) and 64 years (range 43–83) in the observation and radiation therapy group, respectively. The male/female ratio was 27:73 and 15:47 in the observation and radiation therapy groups, respectively. The Chi-square test or Fisher exact test showed significant intergroup differences in terms of age (P < 0.001) and no differences in terms of sex (P = 0.57).

**Table 1:** Demographic Characteristics and Meningioma Information

Variable		Observation group (n = 100)	Treatment group (n = 62)	*P* Value
Patient age (y)*		69 ± 12 (40 ~ 98)	64 ± 10 (43 ~ 83)	< 0.001
No. of men No. of women		27 (27) 73 (73)	15 (24.2) 47 (75.8)	0.57
Follow up period (y)*		2.9 ± 2.5	3.0 ± 2.3	0.87
Post treatment period (y)*			2.6 ± 2.2	0.38
Total radiation dose†		0	1400 (400–2800)	
Location (No.) (%)				< 0.0001
Parasagittal or falx cerebri		76 (76)	32 (51.6)	
Anterior cranial fossa		6 (6)	2 (3.2)	
Cerebellopontine angle		3 (3)	9 (14.5)	
Cerebellar tentorium		3 (3)	1 (1.6)	
Cavernous sinus		2 (2)	3 (4.8)	
Posterior fossa		2 (2)	6 (9.7)	
Olfactory bulb		2 (2)	3 (4.8)	
Lateral ventricle		2 (2)	2 (3.2)	
Middle cranial fossa		2 (2)	1 (1.6)	
Prepontine cistern		1 (1)	0 (0)	
Foramen magnum		1 (1)	0 (0)	
Sella		0 (0)	1 (1.6)	
Straight sinus		0 (0)	1 (1.6)	
Optic sheath		0 (0)	1 (1.6)	
Imaging features (No.) (%)	Description			
CSF cleft	Perimeter of CSF between the tumor and brain	88 (88)	47 (75.2)	< 0.001
Mass effect	Shift in normal brain parenchyma due to tumor	25 (25)	23 (36.8)	0.02
Intratumoral heterogeneity	Heterogeneity in hyperintensity of MRI signal throughout tumor	22 (11)	7 (11.2)	0.87
Skull hyperostosis	Bony overgrowth adjacent to tumor	14 (14)	14 (22.4)	0.07
Multifocality	Non-contiguous growth of tumor	5 (5)	3 (4.8)	0.95
Bony invasion	Appearance of tumor invading the skull	3 (3)	12 (19.2)	< 0.0001
Midline shift	Shift of the brain past midline	3 (3)	3 (4.8)	0.26
Necrosis/Hemorrhage	Presence of necrosis or hemorrhage	3 (3)	5 (8.0)	0.04
Spiculation	Irregularities in tumor shape and border	3 (3)	3 (4.8)	0.39
Cystic component	Fluid filled cysts within the tumor	3 (3)	2 (3.2)	0.76
Sinus invasion	Presence of venous sinus invasion	2 (2)	10 (16)	< 0.0001
Recurrence	Recurrent at presentation	1 (1)	4 (6.4)	0.01

Note: Unless otherwise specified, data are numbers of patients, with percentages in parentheses. Categoric variables were compared using the χ^2^ test or the Fisher exact test, as appropriate. Continuous variables were compared using the Mann-Whitney U test

* Data are expressed as means ± standard deviations; data in parentheses are ranges

† Data are expressed as medians, with ranges in parentheses

The pre-post MRI interval was 2.9 ± 2.5 years (mean ± SD) and 3.0 ± 2.3 years in the observation and treatment groups, respectively. The treatment group post-MRI interval was 2.6 ± 2.2 years (mean ± SD). There were no significant intergroup differences regarding the follow-up (P = 0.87) and post-treatment periods (P = 0.38). In the observation group, meningiomas were found in the following locations: parasagittal or falx cerebri (n = 76), anterior cranial fossa (n = 6), cerebellopontine angle (n = 3), cerebellar tentorium (n = 3), cavernous sinus (n = 2), posterior fossa (n = 2), olfactory bulb (n = 2), lateral ventricle (n = 2), and middle cranial fossa (n = 2), prepontine cistern (n = 1), foramen magnum (n = 1). In the treatment group, meningiomas were observed in the following locations: parasagittal or falx cerebri (n = 32), cerebellopontine angle (n = 9), posterior fossa (n = 6), cavernous sinus (n = 3), olfactory bulb (n = 3), anterior cranial fossa (n = 2), lateral ventricle (n = 2), and cerebellar tentorium (n = 1), middle cranial fossa (n = 1), sella (n = 1), straight sinus (n = 1), optic sheath (n = 1). The latter three locations were not present in the observation group.

Distribution of positions was significantly different between both groups (P < 0.0001). Among the meningioma characteristics, the CSF cleft was well seen in 88 (88%) and 47 (75.2%) patients in the observation and treatment groups, respectively; the difference was statistically significant (P < 0.001). Mass effect was also seen in 23 (36.8%) and 25 (25%) patients in the observation and treatment groups, respectively; the difference was statistically significant (P = 0.02). Bony invasion was observed in three (3%) and 12 (19.2%) patients in the observation and treatment groups, respectively; the difference was statistically significant (P < 0.0001). Necrosis or hemorrhage was observed in three (3%) and five (8%) patients in the observation and treatment groups, respectively; the difference was statistically significant (P = 0.04). Venous sinus invasion was observed in two (2%) and ten (16%) patients in the observation and treatment groups, respectively; the difference was statistically significant (P < 0.0001). Finally, recurrence was observed in one (1%) and four (6.4%) patients in the observation and treatment groups, respectively; the difference was statistically significant (P = 0.01). Additionally, the pre-post intergroup difference in maximal area and diameter of meningiomas was statistically significant (P < 0.0001) (Table 2). However, the pre-post difference of the meningioma SI showed no statistical significance on T1WI, T2WI, FLAIR, CE-FLAIR, and CE-T1WI (Table 2).

**Table 2:** Comparison of area, maximal diameter, MR signal intensity ratio of meningioma between observation and treatment groups

		Pre	Post	P	Difference	P
Area (mm^2^)	Observation group (n = 100)	89.55 (50.70-152.91)	111.02(60.75-218.45)	< 0.0001	16.85 (6.71–42.44)	< 0.0001
	Treatment group (n = 62)	195.07 (124.37-305.69)	195.01 (111.62- 322.04)	0.12	-10.25 (-27.85-16.64)	
Maximal diameter (mm)	Observation group (n = 100)	10.75 (7.18–14.09)	11.72 (8.23–16.17)	< 0.0001	0.91 (0.32–2.36)	< 0.0001
	Treatment group (n = 62)	15.06 (12.18–18.01)	14.65 (11.90- 18.76)	0.12	-0.41 (-1.46 to 0.49)	
T1 signal ratio	Observation group (n = 100)	0.74 (0.59–0.84)	0.67 (0.54–0.82)	0.09	-0.009 (-0.11- 0.05)	0.38
	Treatment group (n = 62)	0.78 (0.62–0.88)	0.71 (0.58–0.84)	0.02	-0.04 (-0.14 to 0.05)	
T2 signal ratio	Observation group (n = 100)	1.29 (1.08 to 1.52)	1.28 (1.01 to 1.45)	< 0.05	-0.07 (-0.18 to 0.05)	0.95
	Treatment group (n = 62)	1.14 (1.21 to 1.56)	1.34 (1.09 to 1.59)	0.04	-0.06 (-0.23 to 0.07)	
CE-T1 signal ratio	Observation group (n = 100)	1.70 (1.42–2.07)	1.67 (1.48–1.99)	0.51	0.009 (-0.23- 0.31)	0.07
	Treatment group (n = 62)	1.88 (1.62–2.16)	1.75 (1.55–2.21)	0.09	-0.09 (-0.34 to 0.16)	
FLAIR signal ratio	Observation group (n = 100)	1.15 ± 0.36	1.08 ± 0.34	0.0061	-0.05 (-0.16-0.07)	0.66
	Treatment group (n = 62)	1.27 ± 0.27	1.19 ± 0.31	0.01	-0.06 (-0.19 to 0.07)	
CE-FLAIR signal ratio	Observation group (n = 100)	1.34 ± 0.46	1.28 ± 0.43	0.13	-0.04 (-0.22 to 0.11)	0.85
	Treatment group (n = 62)	1.51 ± 0.45	1.44 ± 0.41	0.28	-0.03 (-0.21 to 0.18)	

Note: Data are expressed as medians, and data in parentheses are the interquartile range

### Relevant radiomic features (univariate analysis)

**Fig. 3:**
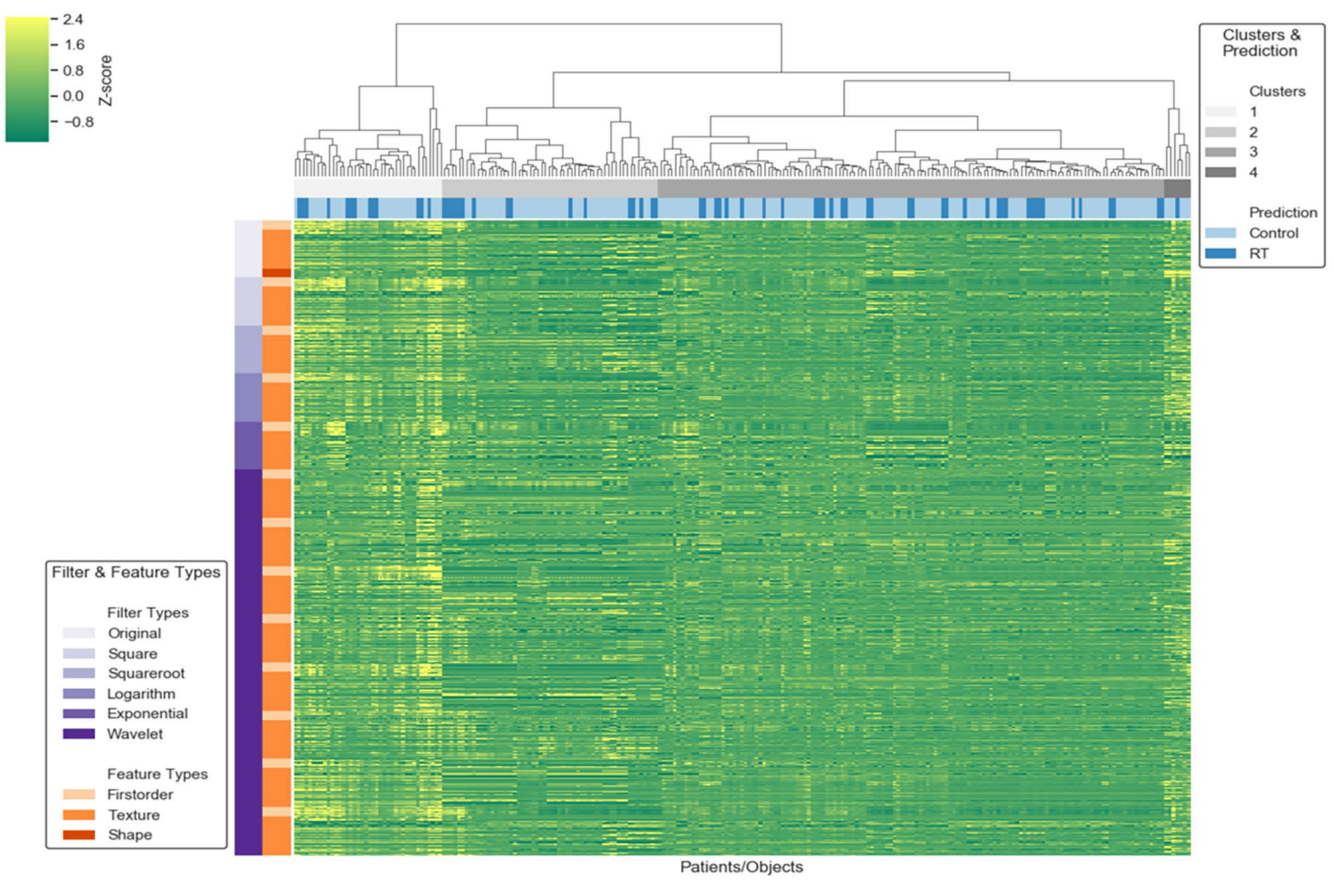
Association test-Clustermap (241 series and 1691 features). Filter Types: Original, Square, Squareroot, Logarithm, Exponential, Wavelet, Feature Types: Firstorder, Texture, Shape

The 162 patients identified for analysis were divided into two cohorts: training (n = 89) and validation sets (n = 73). The training set consisted of 25 males and 64 females (mean age, 67 years; age range, 40–98 years), and the validation set comprised 17 males and 56 females (mean age, 65 years; age range, 43–83 years). Within each ROI in meningioma, the following were extracted from T1WI, T2WI, FLAIR, CE-FLAIR, and CE-T1WI: 19 first-order features, 16 shape-based (3D), 10 shape-shaped (2D), 24 Gy-level co-occurrence matrix features, 16 Gy-level run-length matrix features, 16 Gy-level size-zone matrix features, five neighboring gray tone difference matrix, and 14 Gy-level dependence matrix features. Thus, there were 241 series and 1691 total radiomic features from the original images (Fig. 3). Table 3 demonstrates the most relevant radiomic features for discriminating between the two groups on T1WI, CE-FLAIR, CE-T1WI, and all images (P < 0.05). The most relevant radiomic feature on T1WI was selected as squareroot_firstorder_Kurtosis. We selected the three most relevant radiomic features on CE-FLAIR: one exponential and texture and two square-based features. The 10 most relevant radiomic features on CE-T1WI were selected: two original, four logarithm, and four wavelet-based features. The 10 most relevant radiomic features for the differentiation between both groups on all images were selected: one original, two square, and seven wavelet-based features.

**Table 3:** Radiomic features extracted from brain MRI that were significantly relevant with meningioma of radiation treatment from univariate analysis

Filter & Feature types	Radiomic Features
Original & Shape	original_shape_Compactness2 original_shape_SphericalDisproportion
Square & Texture	square_gldm_SmallDependenceLowGrayLevelEmphasis square_glszm_ZoneEntropy square_glcm_ Idmn
Logarithm & Texture	logarithm_glcm_Idn logarithm_glszm_SmallAreaEmphasis logarithm_ngtdm_Strength
Exponential & Texture	exponential_gldm_SmallDependenceLowGrayLevelEmphasis
Original & Texture	original_glszm_GrayLevelNonUniformityNormalized
First order	squareroot_firstorder_Kurtosis square_firstorder_10Percentile logarithm_firstorder_Skewness wavelet-LLL_firstorder_Kurtosis
Wavelet& Texture	wavelet-LLL_glcm_Idmn wavelet-LHL_glcm_Idmn wavelet-HLH_glcm_MCC wavelet-LLL_glcm_Idmn wavelet-LHL_gldm_DependenceEntropy wavelet-HLL_glcm_Correlation wavelet-HLH_gldm_LargeDependenceEmphasis wavelet-LHL_glrlm_LongRunHighGrayLevelEmphasis wavelet-HHL_glrlm_RunEntropy wavelet-HHH_glszm_SizeZoneNonUniformityNormalized

gldm, gray-level dependence matrix; glszm, gray level size zone matrix; glcm, gray-level co-occurrence matrix; Idmn, inverse difference moment normalized; Idn, inverse difference normalized; ngtdm, neighboring gray tone difference matrix; MCC, maximal correlation coefficient; glrlm, gray level run length matrix

Filter types: Original, Square, Squareroot, Logarithm, Exponential, Wavelet

Feature types: Firstorder, Texture, Shape

**Fig. 4:**
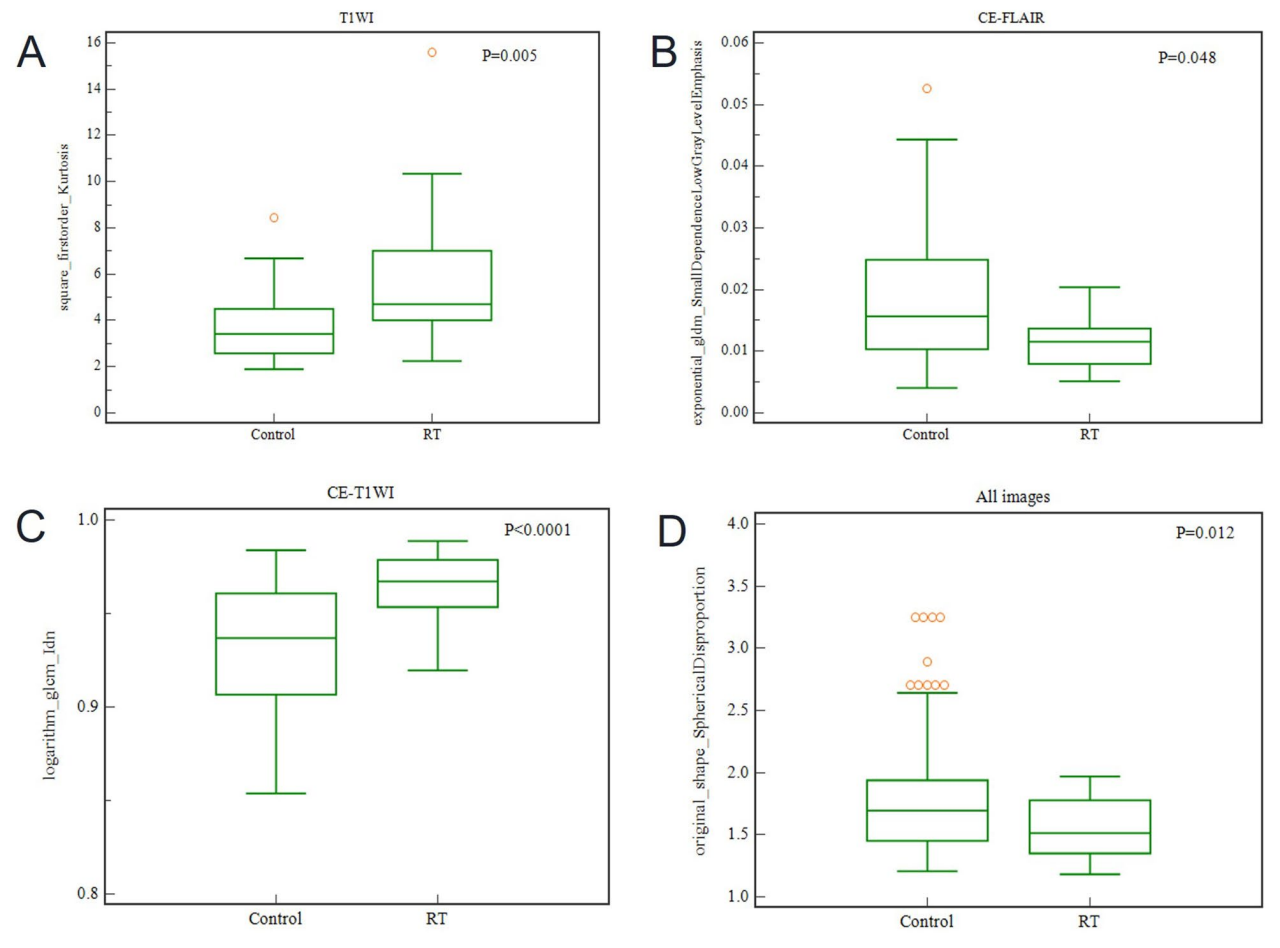
Box plots of the amplitude features, successfully differentiating observation group from radiation treatment group. (**A**) squareroot_firstorder_Kurtosis (P = 0.005), (**B**) exponential_gldm_Small Dependence Low Gray Level Emphasis (P = 0.048), (**C**) logarithm_glcm_Idn (P < 0.0001), (**D**) original_shape_Spherical Disproportion (P = 0.012)

**Fig. 5:**
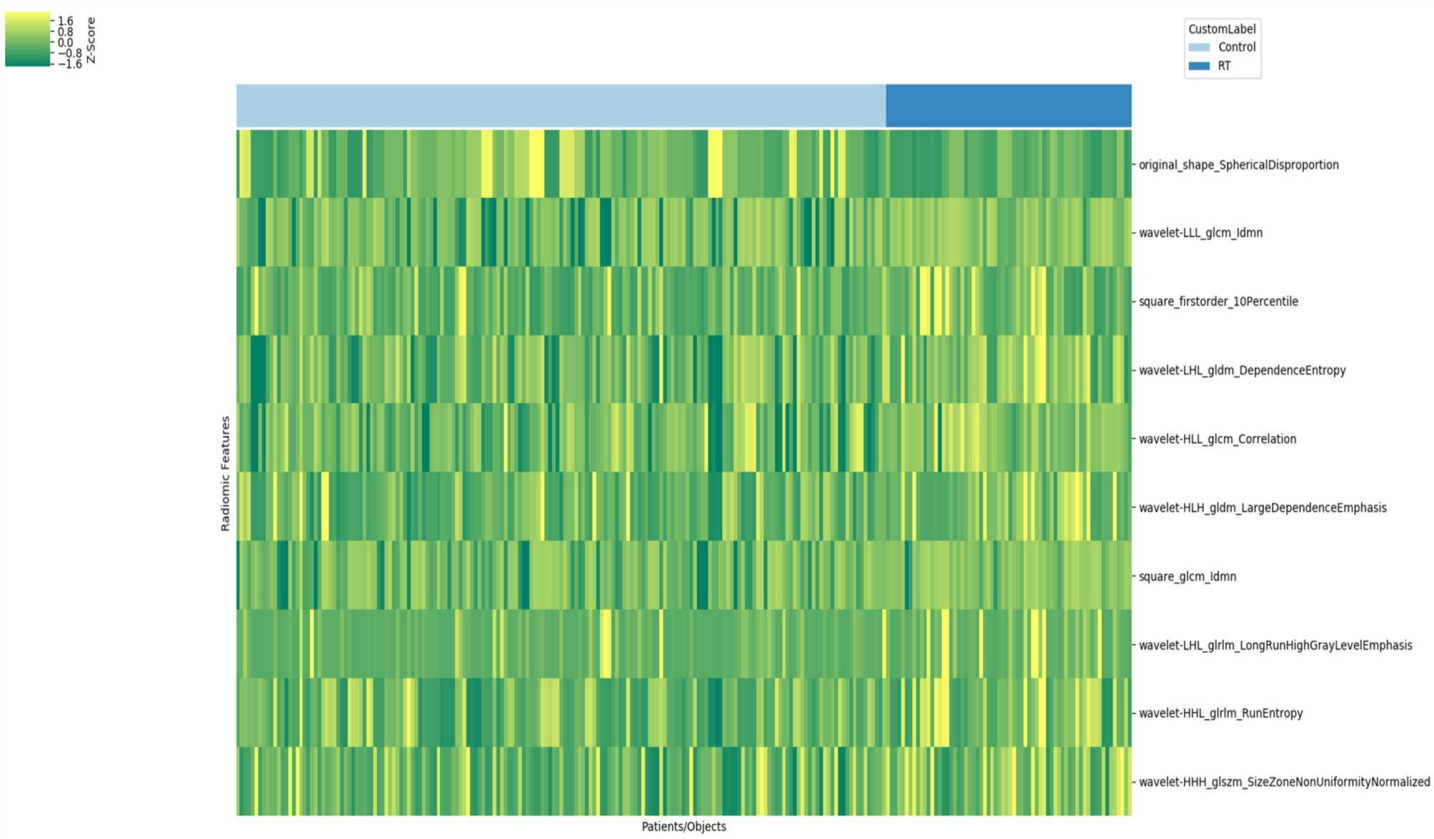
Most relevant features in training set. Feature extraction from MRI and selected 10 most relevant features. One original, two square, and seven wavelet-based features. The heatmap of these radiomic features showed differences between observation and radiation therapy groups

Figure 4 shows box plots of each of the most distinguishable radiomic features from T1WI, CE-FLAIR, CE-T1WI, and all images, successfully differentiating the observation group from the radiation treatment group. The heat map of 10 radiomic features in all images was selected from the variable-hunting algorithm for the random forest model to show the performance of radiomic features extracted from MRI in meningioma after radiation therapy. Each row and column correspond to one normalized radiomic feature and one patient group, respectively (Fig. 5).

### Multivariate results for radiomic features

**Fig. 6:**
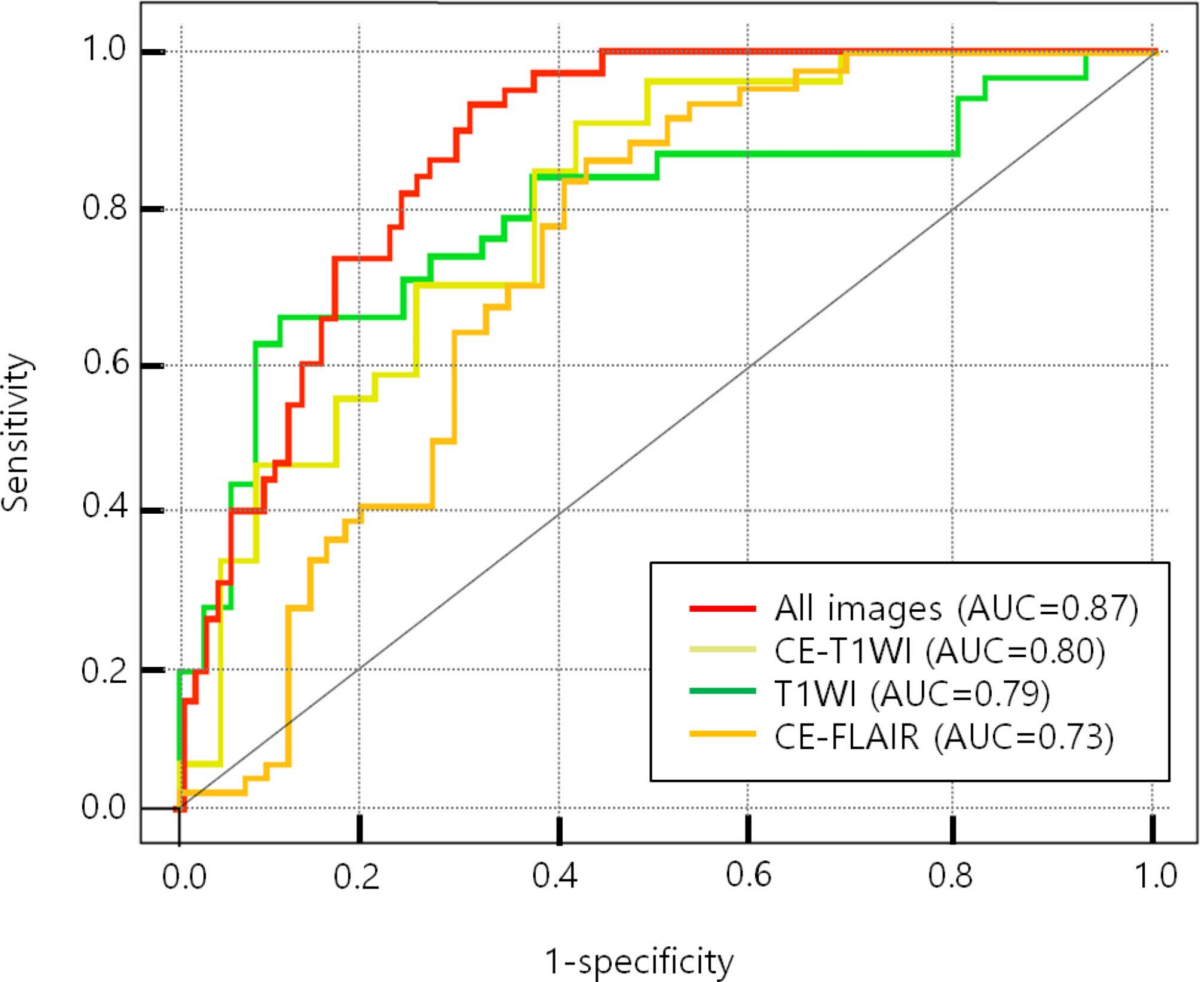
Receiver operating characteristic (ROC) curves based on the significant radiomic features

Table 4; Fig. 6 demonstrate the best combination set of radiomic features for differentiation between both groups on T1WI, CE-FLAIR, CE-T1WI, and all image combinations. One radiomic feature on T1WI was squareroot_firstorder_ kurtosis (OR = 4.45, P < 0.0001), which was significant in their association of meningioma with radiation therapy, with an AUROC of 0.79. The best subset of radiomic features in the training set for differentiating between both groups on CE-FLAIR was exponential_gldm_SmallDependenceLowGrayLevelEmphasis (OR = 4.44, P < 0.0001) had an AUROC of 0.73. The best combination of radiomic features on CE-T1WI was this subset composed of three radiomic features: logarithm_glcm_Idn (OR = 3.385, P < 0.05), wavelet-LLL_firstorder_Kurtosis (OR = 2.049, P < 0.05), original_glszm_GrayLevelNon UniformityNormalized (OR = 1.851, P < 0.05) with an AUROC of 0.80. Five radiomic features on all images were randomly significant in their association with meningioma with radiation therapy (P < 0.05). These features included original_shape_Spherical Disproportion (OR = 2.354, P < 0.05), wavelet-LLL_glcm_Idmn (OR = 2.487, P < 0.05), square_glcm_Idmn (OR = 2.097, P < 0.05), square_firstorder_10Percentile (OR = 1.568, P < 0.05), and wavelet-HLL_glcm_Correlation (OR = 1.825, P < 0.05). The best combination of radiomic features was this subset composed of eight radiomic features with an AUROC of 0.87 of the RF models from 10-fold cross-validation to differentiate between the observation and radiation therapy (Table 4; Fig. 6).

**Table 4:** Logistic regression analysis for best radiomic feature set showing statistical difference between observation and radiation treatment groups in meningioma patients

		Univariate				Multivariate		
Image	Radiomic features	MI	*R* ^*2*^	P	AUC	OR (95% CI)	*P*	AUC
T1WI	squareroot_firstorder_Kurtosis	0.171	0.25	0.005	0.791	4.45 (2.10–9.45)	< 0.0001	0.79
CE-FLALR	exponential_gldm_SmallDependenceLowGrayLevelEmphasis	0.146	0.151	0.048	0.733	4.44 (1.97–10.01 )	< 0.0001	0.73
	square_gldm_SmallDependenceLowGrayLevelEmphasis	0.158	0.142	0.048	0.765			
	square_glszm_ZoneEntropy	0.1	0.14	0.048	0.71			
CE-T1WI	logarithm_glcm_Idn	0.152	0.264	< 0.0001	0.805	3.39 (1.51–7.57)	0.003	0.80
	wavelet-LLL_firstorder_Kurtosis	0.169	0.25	< 0.0001	0.824	2.05 (1.06–3.98)	0.034	
	original_glszm_GrayLevelNonUniformityNormalized	0.082	0.086	0.007	0.639	1.85 (1.10–3.12)	0.021	
	logarithm_firstorder_Skewness	0.178	0.0238	< 0.0001	0.786			
	wavelet-LLL_glcm_Idmn	0.123	0.182	< 0.0001	0.744			
	wavelet-LHL_glcm_Idmn	0.1	0.151	0.0003	0.698			
	original_shape_Compactness2	0.086	0.142	0.0005	0.719			
	logarithm_ngtdm_Strength	0.1	0.125	0.001	0.744			
	logarithm_glszm_SmallAreaEmphasis	0.08	0.073	0.017	0.67			
	wavelet-HLH_glcm_MCC	0.041	0.066	0.026	0.627			
T1WI + T2WI + FLAIR + CE-T1WI + CE-FLAIR	original_shape_SphericalDisproportion	0.067	0.071	0.012	0.669	2.35 (1.21–4.58)	0.012	0.87
	wavelet-LLL_glcm_Idmn	0.062	0.066	0.012	0.662	2.49 (1.24-5.00)	0.010	
	square_glcm_Idmn	0.046	0.051	0.032	0.651	2.10 (1.21–3.64)	0.008	
	wavelet-HLL_glcm_Correlation	0.044	0.055	0.028	0.659	1.83 (1.12–2.98)	0.016	
	square_firstorder_10Percentile	0.043	0.057	0.012	0.601	1.59 (1.04–2.43)	0.033	
	wavelet-HHH_glszm_SizeZoneNonUniformityNormalized	0.036	0.047	0.040	0.638	1.46 (0.99–2.16)	0.060	
	wavelet-HLH_gldm_LargeDependenceEmphasis	0.049	0.052	0.032	0.642	1.42(0.93–2.18)	0.104	
	wavelet-LHL_gldm_DependenceEntropy	0.062	0.056	0.026	0.634	1.59 (0.90–2.82)	0.109	
	wavelet-LHL_glrlm_LongRunHighGrayLevelEmphasis	0.05	0.049	0.037	0.606			
	wavelet-HHL_glrlm_RunEntropy	0.058	0.048	0.040	0.613			

### Validation result

Classification results based on the training group from repeating 10-fold cross-validation are presented in Table 5, including parameters of sensitivity, specificity, precision, F1 score, accuracy, Matthews’s correlation coefficient (MCC), and AUC for the four types of images (T1WI, CE-T1WI, CE-FLAIR, and image combination). Their images achieved higher accuracies of 0.71–0.73 and AUC of 0.70–0.76. In the validation set, relatively high accuracy and AUC were shown in the model application for the difference in assessing meningioma after radiotherapy. All image combinations showed a sensitivity of 78.7%, an accuracy of 0.73, and an AUC of 0.79. The CE-T1WI group showed an accuracy of 0.71 and an AUC of 0.74. There was good agreement between the two independent neuroradiologists in assessing the meningiomas (k = 0.81 [95% CI: 0.78, 0.84]).

**Table 5:** Difference in performance of models of meningioma for observation and treatment groups between training and validation sets

Training/Validation set	Images	Sensitivity (%)	Specificity (%)	Precision	F1 Score	MCC	Accuracy	AUC
Training set	T1WI	51.9	81.5	0.54	0.53	0.34	0.73	0.705
	CE-FLAIR	50	77.3	0.42	0.45	0.26	0.71	0.702
	CE-T1WI	66.7	70.3	0.46	0.54	0.34	0.71	0.749
	T1WI + T2WI + FLAIR + CE-T1WI + CE-FLAIR	66	75.9	0.71	0.74	0.52	0.71	0.758
Validation set	T1WI	53.3	83.5	0.61	0.57	0.30	0.75	0.713
	CE-FLAIR	58.1	76.9	0.69	0.71	0.33	0.71	0.721
	CE-T1WI	63.6	73.7	0.48	0.54	0.37	0.71	0.739
	T1WI + T2WI + FLAIR + CE-T1WI + CE-FLAIR	78.7	67.4	0.71	0.75	0.46	0.73	0.787

MCC: Matthews Correlation Coefficient

## Discussion

Radiation therapy has developed in recent years, and many new techniques have emerged [14].Techniques capable of increasingly accurate tumor localization have been developed to minimize the exposure of the normal brain to high radiation [15]. Radiation therapy is widely used for treating meningiomas that are difficult to access surgically, such as those in the skull base and optic pathway [16]. There are reports that radiation therapy for local control after subtotal excision can improve the 5-year progression-free survival by up to 95% [16]. Radiation therapy after subtotal excision is effective enough. A study tracked 84 meningiomas treated with stereotactic radiosurgery; the tumor volume was reduced by 33% after 24 months and 36% after 36 months [17, 18]. In our study, as a result of follow-up for approximately 3 years, the size and maximal diameter increased significantly in the observation group. However, growth was not evident or slightly decreased in the radiation treatment group. There was a significant difference in meningioma size between both groups. Conversely, there was no significant difference in the SI and contrast enhancement of meningioma between both groups after radiotherapy. This result is considered meaningful.

Both groups differed significantly regarding age and meningioma location. Patients in the radiation therapy group were younger, and meningiomas in the observation group were mostly located in the parasagittal or falx cerebri. Our study showed that radiation therapy was used more frequently in treating meningiomas that are difficult to access surgically. Examples are those located in the cerebellopontine angle, cerebellar tentorium, cavernous sinus, posterior fossa, olfactory bulb, sella, straight sinus, and optic sheath. Significantly more findings, such as CSF cleft, mass effect, bony invasion, necrosis/hemorrhage, venous sinus invasion, and recurrence, were observed in the radiation treatment group than in the observation group.

In this study, 2 patients in the radiation treatment group increased rapidly in tumor size even after radiotherapy. After surgery, the meningioma was confirmed as atypical. The possibility of atypical or malignant meningioma cannot be completely ruled out if there is a rapid increase in size after radiation therapy [19]. To date, no paper has studied the discrimination of radiomic features after radiation treatment of meningioma based on MRI. This study is the first to propose a radiology model based on brain MRI.

In our study, we could not extract meaningful radiomic features from T2WI and FLAIR, but we extracted radiomics of T1WI, CE-FLAIR, CE-T1WI, and five image combinations. A total of 24 radiomic features showed a high correlation with radiation treatment, and the combination of images showed good predictive power in training (AUC: 0.76) and validation (AUC: 0.79). The single images T1WI, CE-FLAIR, and CE-T1WI showed relatively high predictive power comparable to the combination of images.

We investigated the correlation between radiomic features and radiation therapy. Among 1595 radiomic features, 14 showed a high correlation in T1WI, CE-FLAIR, and CE-T1WI. Many of these features were textural features of the image, features measuring gray-level values, and radiomic features measuring heterogeneity in the texture patterns, local homogeneity, or heterogeneity of the image. These features showed the microscopic descriptions of the meningioma. These features were not easily discernible to the human eye or understood or interpretable in any meaning [20–24]. Here, the radiomic feature called CE-FLAIR_Small Dependence Low Gray Level Emphasis was a meaningful element commonly derived from univariate and multivariate analysis results. Radiomic features emphasizing gray level were significantly associated with brain invasion of meningiomas [20]. CE-T1WI original_shape_Compactness2, orginal_shape_Spherical Disproportion, and logarithm-glasm small area emphasis seemed to show changes in tumor size and shape. The values of these features were lower in the radiation treatment group than in the observation group, suggesting that changes in meningioma size and shape are related to radiation therapy. A study that investigated the association between radiomic and semantic features of meningioma reported that spherical disproportion was related to mass effect, speculation, and bony and venous sinus invasion [1].

Kurtosis, a radiomic feature commonly seen in T1WI and CE-T1WI, is a measure of the peakedness of the distribution of values in meningioma ROI, meaning that higher values do not converge to the mean but spread toward the tail of a normal distribution [25]. Radiomic features reflect the microscopic heterogeneity within tumors associated with radiation therapy. They are a new tool for predicting meningioma changes after radiation therapy. It is relatively easy to compare the change in SI of the image by measuring the ROI. However, it was a very interesting task as it provided information on the changes in the radiomic features of meningioma after radiation treatment.

Our results suggest that the radiomic features of meningiomas can be used to predict changes and after radiotherapy. This study attempted to extract radiomic features from each image by selecting T1WI, T2WI, FLAIR, CE-FLAIR, and CE-T1WI. CE-T1WI is commonly used to define macroscopic tumor boundaries and evaluate the extent of tumor invasion and blood supply [26]. T2 imaging is sensitive to watery tissues and can be used to detect the presence of edema [27]. Other studies have introduced features related to meningioma and brain invasion in T2WI [20].

However, this study showed no significant difference between the observation and treatment groups in the SI ratio in T2WI and FLAIR. Radiomic features also had no meaningful extracted information. This seems to require more in-depth research with more patients. In this study, the multiple sequence models (T1WI, T2WI, FLAIR, CE-T1WI, and CE-FLAIR combined) showed better predictive power than the single models. These results suggest that multiple sequences may provide more information about the tumor and better show radiation-related changes in meningiomas.

Our study had several limitations. First, as this was a retrospective study, histological confirmation was not performed in all patients. Meningioma removal surgery was performed on a limited basis only in some cases with enlarged tumors. Although evaluated using imaging, image evaluation may be subjective. Second, this study was conducted at a single institution with a limited sample size. Further studies with larger sample sizes from multiple centers are required for external validation. External validation is needed to evaluate the reproducibility and optimize the model. Third, there may be an unavoidable selection bias, as approximately 18% of the patients were excluded from the training and validation cohorts for various reasons. Fourth, MRI scans were retrospectively collected on three MRI machines with different devices and acquisition parameters, and radiomic features are sensitive to parameters. Therefore, it is necessary to normalize the MR images to obtain a standard normal distribution of image intensities. Finally, in the T1WI, T2WI, and FLAIR sequences, the meningioma and surrounding borders were unclear in some cases. We referenced the CE-T1WI sequence for visual guidance; however, deviations remained. In the future, multimodal studies, such as DWI and ADC map sequences, can be integrated into the model to further improve accuracy.

In conclusion, this study evaluated radiomic features and radiologic appearances of meningiomas, which were significantly different after radiotherapy. Our findings can help predict size reduction after radiotherapy for meningioma. A radiomic model using MR images can be useful as a biomarker to predict changes in meningiomas after radiation treatment. This is expected to have a positive effect on patient treatment.

### Electronic supplementary material

Below is the link to the electronic supplementary material.

Supplementary Material 1

## Data Availability

The datasets generated and/or analyzed during the current study are not publicly available due to local ownership of the data but are available from the corresponding author on reasonable request.
